# The Akt-mTOR axis is a pivotal regulator of eccentric hypertrophy during volume overload

**DOI:** 10.1038/srep15881

**Published:** 2015-10-30

**Authors:** Masataka Ikeda, Tomomi Ide, Takeo Fujino, Yuka Matsuo, Shinobu Arai, Keita Saku, Takamori Kakino, Yasuhiro Oga, Akiko Nishizaki, Kenji Sunagawa

**Affiliations:** 1 Department of Cardiovascular Medicine, Graduate School of Medical Sciences, Kyushu University, Fukuoka, Japan

## Abstract

The heart has two major modalities of hypertrophy in response to hemodynamic loads: concentric and eccentric hypertrophy caused by pressure and volume overload (VO), respectively. However, the molecular mechanism of eccentric hypertrophy remains poorly understood. Here we demonstrate that the Akt-mammalian target of rapamycin (mTOR) axis is a pivotal regulator of eccentric hypertrophy during VO. While mTOR in the heart was activated in a left ventricular end-diastolic pressure (LVEDP)-dependent manner, mTOR inhibition suppressed eccentric hypertrophy and induced cardiac atrophy even under VO. Notably, Akt was ubiquitinated and phosphorylated in response to VO, and blocking the recruitment of Akt to the membrane completely abolished mTOR activation. Various growth factors were upregulated during VO, suggesting that these might be involved in Akt-mTOR activation. Furthermore, the rate of eccentric hypertrophy progression was proportional to mTOR activity, which allowed accurate estimation of eccentric hypertrophy by time-integration of mTOR activity. These results suggested that the Akt-mTOR axis plays a pivotal role in eccentric hypertrophy, and mTOR activity quantitatively determines the rate of eccentric hypertrophy progression. As eccentric hypertrophy is an inherent system of the heart for regulating cardiac output and LVEDP, our findings provide a new mechanistic insight into the adaptive mechanism of the heart.

The heart is a vital organ that maintains homeostasis in the body via blood circulation. To maintain blood circulation in the peripheral tissues, the heart is capable of remodeling in response to various stresses including hemodynamic load, neurohormones, oxidative stress, and cytokines^1^,^2^,^3^. Among those, mechanical loads are major inputs for the heart because the heart is incessantly subject to hemodynamic stresses. Given that mechanical load induces hypertrophy, mechanical stretching forces in systole and diastole in myocytes in *vivo* can be calculated as wall stresses in systole and diastole in accordance with Laplace’s law. In 1975, Grossman *et al.* demonstrated that pressure overload (PO) increases systolic wall stress, resulting in concentric hypertrophy, which in turn normalizes systolic wall stress, and that volume overload (VO) increases diastolic wall stress, resulting in eccentric hypertrophy^1^. Based on this clinical observation, they proposed that the hypertrophic response was evoked by increased wall stress^1^. Currently, it is widely accepted that increased systolic and diastolic wall stresses lead to concentric and eccentric hypertrophy, respectively^4^. Many lines of evidence indicate that concentric and eccentric hypertrophy differ not only in terms of phenotype but also in the intracellular signaling pathways that are involved^5^,^6^. Various studies investigated the molecular mechanism of hypertrophy, especially in concentric hypertrophy caused by PO^7^,^8^; however, the molecular mechanism of eccentric hypertrophy has yet to be fully elucidated.

Mitral and aortic regurgitation are typical pathophysiologies that cause VO, especially in the acute phase^9^. In those pathophysiologies, insufficient net forward output inevitably induces pulmonary congestion resulting from increased left ventricular end-diastolic pressure (LVEDP) and diastolic wall stress to the left ventricle (LV). Eccentric hypertrophy increases cardiac output and makes it possible to maintain the net forward output in the presence of regurgitation, thereby lowering LVEDP and resolving pulmonary congestion^9^. Hence, it appears that diastolic wall stress, as defined by LVEDP and LV geometry, triggers eccentric hypertrophy as a physiologically well-designed feedback system for regulating LVEDP.

Additionally, in heart failure (HF), the heart is exposed to excessive diastolic wall stress regardless of the etiology, as HF is a syndrome characterized by low cardiac output and pulmonary congestion (due to high LVEDP). Therefore, the understanding of the as yet-to-be elucidated molecular mechanism of eccentric hypertrophy during VO is important to fully understand the pathophysiology of HF.

Grant *et al.* and Grossman *et al.* proposed that, in terms of physiology, eccentric hypertrophy is analogous to normal cardiac growth, as both processes increase cardiac output, indicating that the structural changes of eccentric hypertrophy and cardiac growth share a common mechanism^1^,^10^. The phosphoinositide-3 kinase/protein kinase B/mammalian target of rapamycin (PI3K/Akt/mTOR) signaling pathway plays an important role in cell and organ growth^11^. mTOR was identified in the early 1990s^12^,^13^ and is the core protein of two functionally distinct complexes, which are called mTOR complex 1 (mTORC1) and mTOR complex 2 (mTORC2)^11^,^14^. mTORC1 is activated by various signals including insulin, growth factors, calcium, and amino acids, through receptors such as receptor of tyrosine kinases and G-protein-coupled receptors^15^,^16^. mTORC1 functions as a serine/threonine kinase and phosphorylates p70S6K at Thr389 and 4E-BP at Thr37/46, and Ulk-1 at Ser757, thereby regulating protein synthesis and macro-autophagy, respectively^17^,^18^. mTORC1 regulates cell growth through protein synthesis, and it has been reported that mTOR and Hippo pathways collaborate to determine organ size^19^,^20^. Therefore, we hypothesized that eccentric hypertrophy is regulated by mTORC1 activation in response to diastolic wall stress.

In the field of cardiology, Sadoshima and Izumo first described the role of mTOR in angiotensin II-induced hypertrophy in myocytes^21^. In addition, mTOR was shown to be involved in PO-induced hypertrophy and LV remodeling after myocardial infarction in mice^22^,^23^. While these studies indicated that mTOR plays a key role in the physiology and pathology of the heart^24^, the precise mechanism of mTOR regulation in the heart is still unknown.

In this study, we demonstrated that the Akt-mTOR axis regulates eccentric hypertrophy during VO in response to diastolic wall stress and that the mTOR activity determines the rate of eccentric hypertrophy progression, by showing that the heart weight (HW) during VO can be accurately estimated by time integration of mTOR activity.

## Results

### Evaluation of the physiological parameters during VO in mice

**Figure 1:**
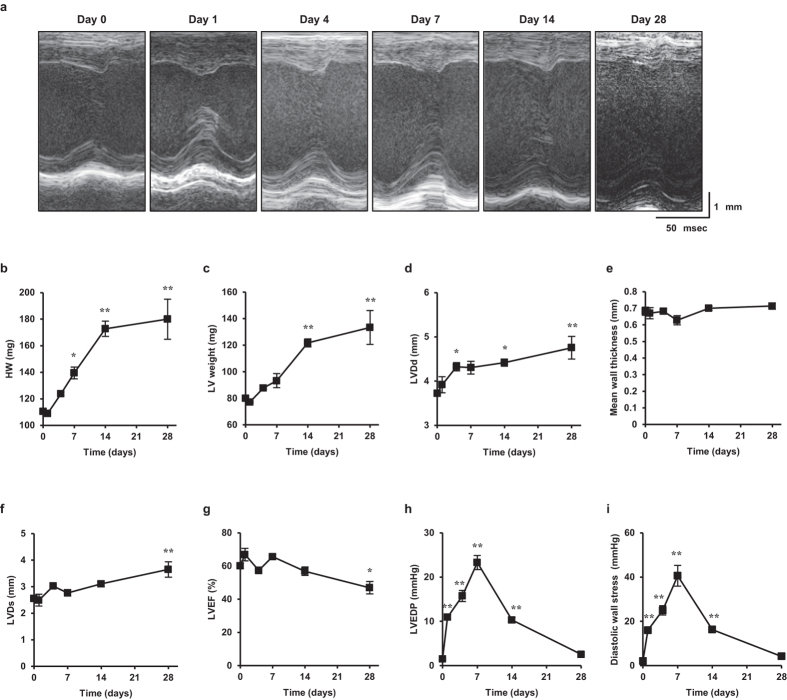
Cardiac hypertrophy, function, and hemodynamics during VO after AVF creation. (**a**) Echocardiogram of VO after AVF creation. (**b**–**i**) heart weight (HW) (**b**), left ventricular (LV) left ventricular (LV) weight (**c**), left ventricular diameter in diastole (LVDd) (**d**), mean wall thickness (average of the interventricular septum and the left ventricular posterior wall) (**e**), left ventricular diameter in systole (LVDs) (**f**), left ventricular ejection fraction (LVEF) (**g**), left ventricular end diastolic pressure (LVEDP) (**h**), diastolic wall stress (**i**) during the 4-week time course of VO. Data are shown as the mean ± SEM (n = 4). *P < 0.05, **P < 0.01 vs. day 0 (Dunnett’s test).

We first established an eccentric hypertrophy model accompanied by an increase in heart and LV mass and LV dilatation by creating an arteriovenous fistula (AVF) in the abdominal aorta, without any detectable alternation in the mean wall thickness (Fig. 1a–e). In this model, cardiac hypertrophy increased steadily until day 28, with a mild but significant increase of left ventricular diameter in systole (LVDs) and impairment of the left ventricular ejection fraction (LVEF) (Fig. 1f,g). The LVEDP and diastolic wall stress reached a peak on day 7 and decreased thereafter, returning to baseline values by day 28 (Fig. 1h,i). The initial increase in the LVEDP until day 7 may be caused by compensatory volume retention to preserve the systemic pressure, especially in the kidneys, while the normalization of the LVEDP and diastolic wall stress after day 7 was consistent with the time course of chronic VO caused by mitral or aortic regurgitation in humans^9^. It is worth noting that VO did not change heart rate or peak pressure (Supplementary Fig. S1a,b), indicating that the hemodynamic stress in this VO model was exclusively due to diastolic wall stress. These results suggested that the eccentric hypertrophy resulting from diastolic wall stress during VO is a physiological feedback system for regulating the LVEDP that buffers an excessive preload via LV dilatation.

### mTOR activity is dependent on diastolic wall stress during VO

**Figure 2:**
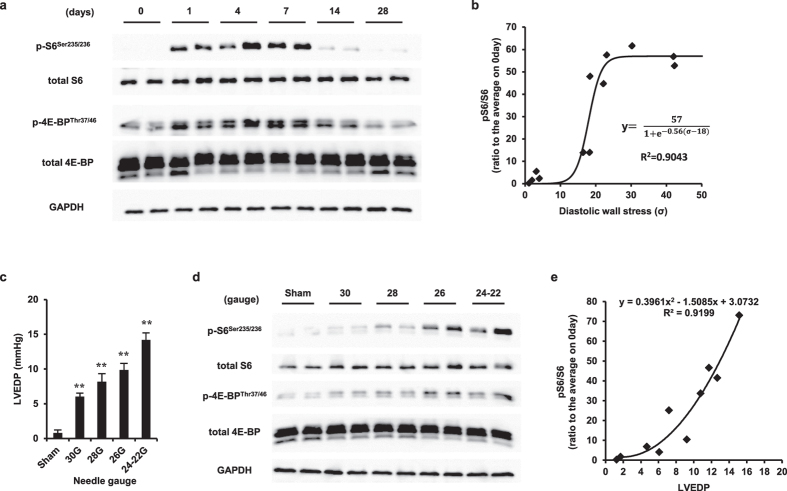
Relationship between LVEDP and mTOR activity. (**a**) Western blot of cell lysates obtained from heart tissue during 4 weeks of VO, probed for total and phosphorylated S6 and 4E-BP. (**b**) Relationship between diastolic wall stress and mTOR activity during 4 weeks of VO. (**c**) Left ventricular end diastolic pressure (LVEDP) at 24 h after induction of VO by using various needle sizes to penetrate the vein/artery (n = 3). (**d**) Western blot of cell lysates obtained from heart tissue at 24 h after VO induction using various needle sizes to penetrate the artery, probed for total and phosphorylated S6 and 4E-BP. (**e**) Relationship between LVEDP and S6 phosphorylation rate at 24 h after VO induction. GAPDH was used as a loading control. Data are shown as the mean ± SEM. **P < 0.01 vs. sham (Dunnett’s test).

Eccentric hypertrophy involves structural alterations of the heart that increase cardiac output and that are physiologically similar to those occurring during cardiac growth, in which the PI3K/Akt/mTOR signaling pathway plays an important role. Therefore, the role of mTORC1 in mediating eccentric hypertrophy was investigated by assessing the phosphorylation status of S6 and 4E-BP. The increased phosphorylation of the two proteins indicated that the mTOR activity increased in a diastolic wall stress-dependent manner during VO (Fig. 2a). The relationship between diastolic wall stress and the phosphorylated S6/S6 ratio followed a sigmoidal curve (R^2^ = 0.90) (Fig. 2b). To exclude the possibility that the prolonged stimulus altered the signal transduction in mechanical stretch-induced mTOR activation during the late phase, mTOR activity in the myocardium was evaluated 24 h after AVF creation. The heart was subjected to different diastolic loads at 24 h by altering the shunt size using penetrating needles of various sizes. As expected, LVEDP elevation depended on the shunt size (Fig. 2c), and S6 and 4E-BP phosphorylation, i.e., mTOR activation, was correlated with the LVEDP (Fig. 2d). The phosphorylated S6/S6 ratio was well quadratically correlated with LVEDP (R^2^ = 0.92) (Fig. 2e). These results suggested that diastolic wall stress governs the mTOR activity.

### mTOR is a pivotal regulator of eccentric hypertrophy during VO

**Figure 3:**
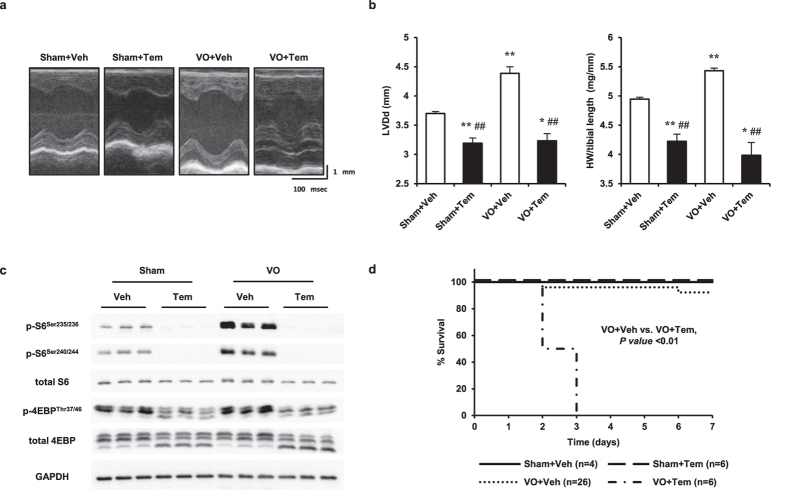
Effect of mTOR inhibition on eccentric hypertrophy during VO. (**a**) Echocardiogram of AVF- or sham-operated mice treated with temsirolimus (Tem) or vehicle (Veh) on day 3 after AVF creation. (**b**) LVDd (left panel) and HW/tibial length (right panel) on day 3 of VO (n = 3–4). (**c**) Western blot of cell lysates obtained from heart tissue of AVF- or sham-operated mice treated with Tem or Veh on day 3 of VO, probed for total and phosphorylated S6 and 4E-BP. GAPDH was used as a loading control. (**d**) Survival analysis of AVF- or sham-operated mice treated with Tem or Veh over 7 days of VO. Data are shown as the mean ± SEM. *P < 0.05, **P < 0.01 vs. sham, ^#^*P* < 0.05, ^##^*P* < 0.01 vs. VO + Veh (one-way ANOVA). Survival analysis was performed by the Kaplan-Meier method, and between-group differences in survival were evaluated with the log-rank test.

To clarify the role of mTOR in eccentric hypertrophy, we investigated the effects of temsirolimus, an allosteric inhibitor of mTOR and a rapamycin derivative, on eccentric hypertrophy. mTOR inhibition not only suppressed eccentric hypertrophy but also induced cardiac atrophy, which was characterized by a decrease in the LV diameter in diastole (LVDd) and HW, along with S6 and 4E-BP dephosphorylation (Fig. 3a–c). Interestingly, the survival rate under VO was significantly lower in the temsirolimus-treated than in the vehicle-treated group, while LVDs and LVEF were preserved in all groups (Fig. 3d, Supplementary Fig. S2). These results suggested that mTOR is a pivotal regulator of eccentric hypertrophy during VO and that eccentric hypertrophy is essential for survival under acute VO.

In addition, the atrophic effect of mTOR inhibition on established eccentric hypertrophy was also examined. Mice operated for AVF were randomized into temsirolimus- and vehicle-treated groups at 4 weeks after AVF creation and were administered temsirolimus or vehicle for 1 week (Supplementary Fig. S3a). mTOR inhibition reduced the LVDd and HW, suggesting that it induced cardiac atrophy in the established eccentric hypertrophy (Supplementary Fig. S3b–c).

### Phosphorylation and K63 ubiquitination of Akt are associated with diastolic wall stress during VO

**Figure 4:**
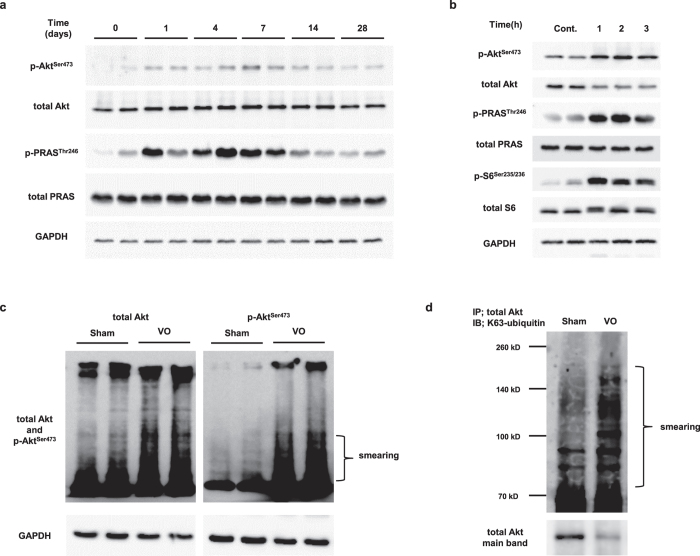
Phosphorylation and ubiquitination of Akt in response to VO. (**a**) Western blot of cell lysates obtained from heart tissue during 4 weeks of VO, probed for total and phosphorylated Akt and PRAS. (**b**) Western blot of cell lysates obtained from the heart tissue during 3 h of VO, probed for the indicated Akt-mTOR signaling pathway components. (**c**) Total and phosphorylated Akt expression in heart tissue after 2 h of VO (with long exposure time). The smearing of the protein band suggests posttranslational modification of Akt residues. (**d**) Immunoblot (IB) of K63-ubiquitin in immunoprecipitates (IP) of heart tissue lysates detected with an antibody against Akt. GAPDH was used as a loading control.

Akt is the best-known kinase mediating mTOR activation and is phosphorylated during VO^6^. Our results revealed that phosphorylation of Akt at Ser^473^ and of PRAS in response to diastolic wall stress was consistent during VO (Fig. 4a). Furthermore, phosphorylation of Akt and Akt downstream signaling (PRAS and S6) was found to occur 1–3 h after AVF creation (Fig. 4b). These results suggest that Akt is involved in mTOR activation under VO.

The immunoblot analysis further revealed a slight decrease in the total Akt level under VO (Fig. 4b), suggesting that Akt may be subject to posttranslational modification under these conditions. With longer exposure of the membrane, smearing of the Akt band in the diastolic loaded heart samples was observed for both total and phosphorylated Akt, indicating posttranslational modification of Akt residues (Fig. 4c).

In addition to phosphorylation, posttranslational modification of Akt is known to include O-GlcNAcylation and ubiquitination^25^,^26^,^27^. K63-linked ubiquitination is required for membrane recruitment and subsequent phosphorylation of Akt. Therefore, we examined the K63 ubiquitination of Akt during VO and found that Akt was modified by K63 ubiquitination under VO (Fig. 4d). Taken together, these results indicated that both K63 ubiquitination and phosphorylation of Akt might play a key role in mTOR activation during VO.

### Akt is a major mediator of diastolic wall stress-mediated mTOR activation during VO

**Figure 5:**
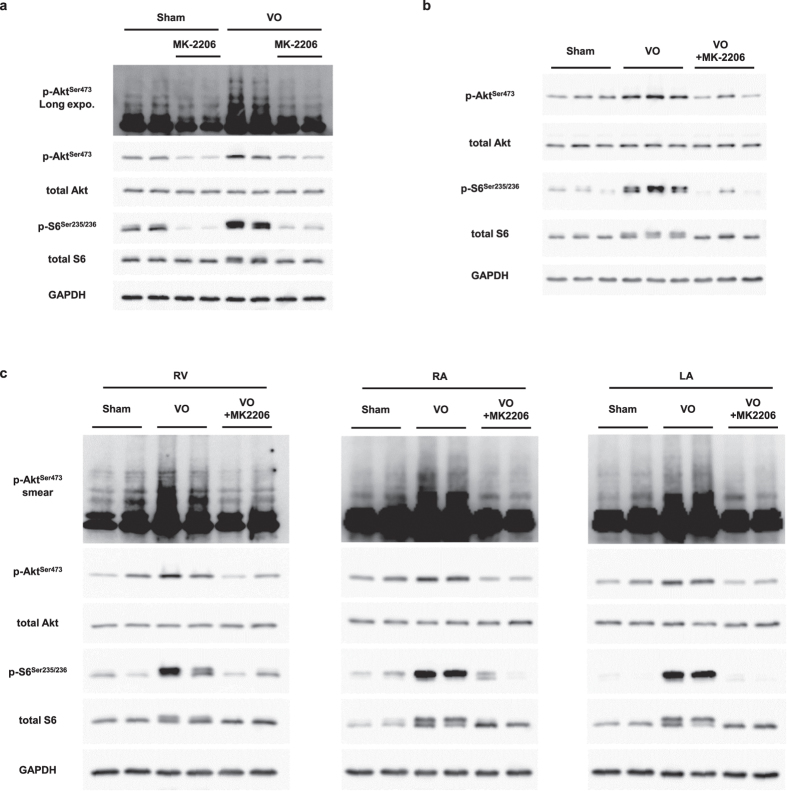
Role of Akt in mTOR activation during VO. (**a**) Western blot of cell lysates obtained from heart tissue after 2 h of VO following 6 h of MK-2206 pre-treatment, probed for total and phosphorylated Akt and S6 (with long membrane exposure time). (**b**) Western blot analysis of total and phosphorylated Akt and S6 8 h after MK-2206 administration on day 3 of VO. (**c**) Western blot of cell lysates obtained from the right ventricle (RV) and right and left atria (RA and LA, respectively) on day 3 of VO, probed for total and phosphorylated Akt and S6. GAPDH was used as a loading control.

To evaluate whether Akt mediates diastolic wall stress via mTOR activation, we used the allosteric inhibitor MK-2206, which targets the pleckstrin homology (PH) domain and prevents the recruitment of Akt to the cell membrane^28^,^29^. MK-2206 has been proven to have high specificity for Akt and much lower effect on mTOR and p70S6 K^30^. Akt phosphorylation was first observed 10 min after AVF creation (Supplementary Fig. S4a) and persisted until 3 h after AVF creation (Fig. 4b). In mice that were orally administered MK-2206 (120 mg/kg), Akt and S6 phosphorylation were inhibited at 4–16 h and at 8–20 h after administration, respectively (Supplementary Fig. S4b). Therefore, the AVF was created 6 h after MK-2206 administration and myocardium samples were collected 2 h later after AVF creation. Phosphorylation of both ubiquitinated and non-ubiquitinated Akt was inhibited by MK-2206 treatment, and S6 phosphorylation was concomitantly suppressed, suggesting that ubiquitination, membrane recruitment, and phosphorylation of Akt are necessary for mTOR activation under VO (Fig. 5a). Furthermore, mTOR activation on day 3 of VO was completely inhibited by MK-2206 (Fig. 5b). These results suggested that Akt is a key mediator of mechanical stress-induced mTOR activation during VO.

In the VO model used in this study, AVF induced VO not only in the left ventricle, but also in the right and left atria, and in the right ventricle. Therefore, we investigated Akt-mTOR signaling in these chambers and found that Akt-mTOR signaling was activated during VO and fully blocked by MK-2206, indicating that the role of Akt-mTOR signaling in eccentric hypertrophy during VO is common to all chambers of the heart (Fig. 5c).

### Diastolic wall stress induces the upregulation of various growth factors during VO

**Figure 6:**
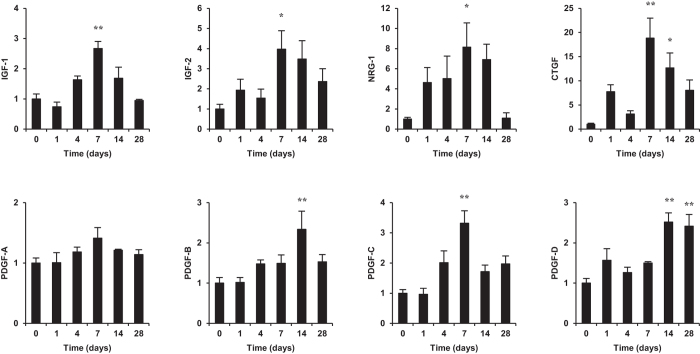
Gene expression of various growth factors during VO. IGF, NRG, CTGF, and PDGF expression was quantified by RT-qPCR and normalized to the expression of the 18S rRNA gene. Data are shown as the mean ± SEM (n = 4). *P < 0.05, **P < 0.01 vs. day 0 (Dunnett’s test).

To identify activators of Akt, the expression of various growth factors including insulin-like growth factor (IGF)-1 and -2, neuregulin (NRG)-1, connective tissue growth factor (CTGF), and platelet-derived growth factor (PDGF)-A, -B, -C, and -D was examined. The expression of NRG-1, IGF-2, and CTGF was upregulated on day 1 after AVF creation, that of IGF-1, IGF-2, NRG-1, CTGF, and PDGF-C was significantly upregulated on day 7, and that of CTGF, PDGF-B, and PDGF-D on day 14 (Fig. 6). These results suggested the possibility that several growth factors are involved in Akt-mTOR activation during VO.

### Accurate estimation of HW by time integration of mTOR activity during VO

mTOR is known as the rate-limiting kinase for cell and organ growth^19^,^20^. We curve-fitted the trend of HW during VO using Excel software (Supplementary Fig. S5a), and resampled 28 points from the fitted curve using Graphcel v1.11 (Supplementary Fig. S5b). We next calculated the difference between consecutive resampled points (Supplementary Fig. S5c), which corresponded to the rate of eccentric hypertrophy progression per day. This detailed analysis revealed that the trend of the progression rate during VO is similar to that of each average (n = 2) of mTOR activity (Supplementary Fig. S5d), suggesting that mTOR activity lineally determines the rate of eccentric hypertrophy progression. Therefore, we hypothesized that the rate of increase in HW (*v* mg/unit time) during VO is proportional to mTOR activity as *v* = α × mTOR activity (where α is an appropriate constant). Thus, the time integration of *v* yields HW as follows:


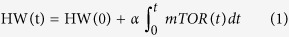


where HW(0) is the initial HW (=110) and mTOR (t) is time-varying mTOR activity.

**Figure 7:**
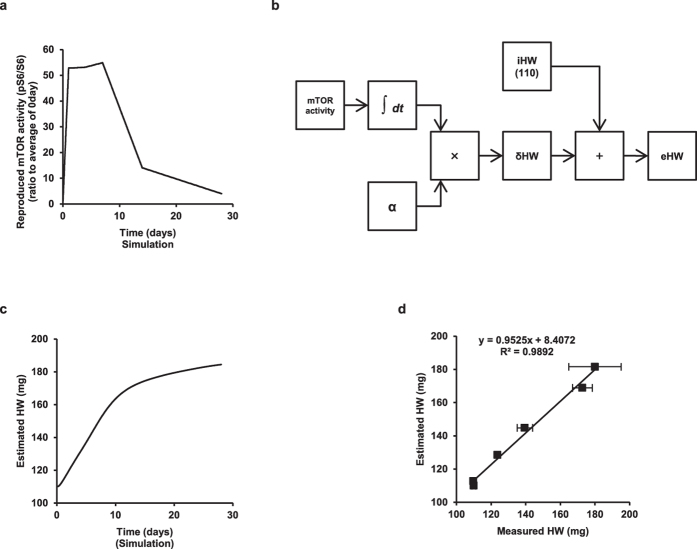
Estimation of eccentric hypertrophy progression during VO. (**a**) Reproduced curve of mTOR activity during VO from resampled data points. (**b**) Schematic diagram to estimate HW from the time series of mTOR activity (pS6/S6 ratio) during VO. (**c**) Estimated HW during VO when α = 0.001. (**d**) Correlation between measured and estimated HW. Data are shown as the mean ± SEM (n = 4).

We plotted the points in each average of mTOR activity (n = 2) during VO in a graph and connected each point, as shown in Supplementary Fig. S5d, and 100 data points per day were resampled. The resampled data accurately reproduced the experimentally measured mTOR activity (Fig. 7a). We solved equation (1) with the Simulink program (Fig. 7b). The estimated HW curve approximated the experimentally measured HW curve well (Fig. 1b and Fig. 7c). Furthermore, the absolute values of the estimated HW agreed well with those measured, if the appropriate constant (α = 0.001) was given (Fig. 7d). These results provided strong evidence that mTOR activity is proportional to the rate of eccentric hypertrophy progression during VO.

Moreover, we found that the mathematical relationship between diastolic wall stress (σ) and mTOR activity was as follows (Fig. 2b):


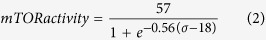


**Figure 8:**
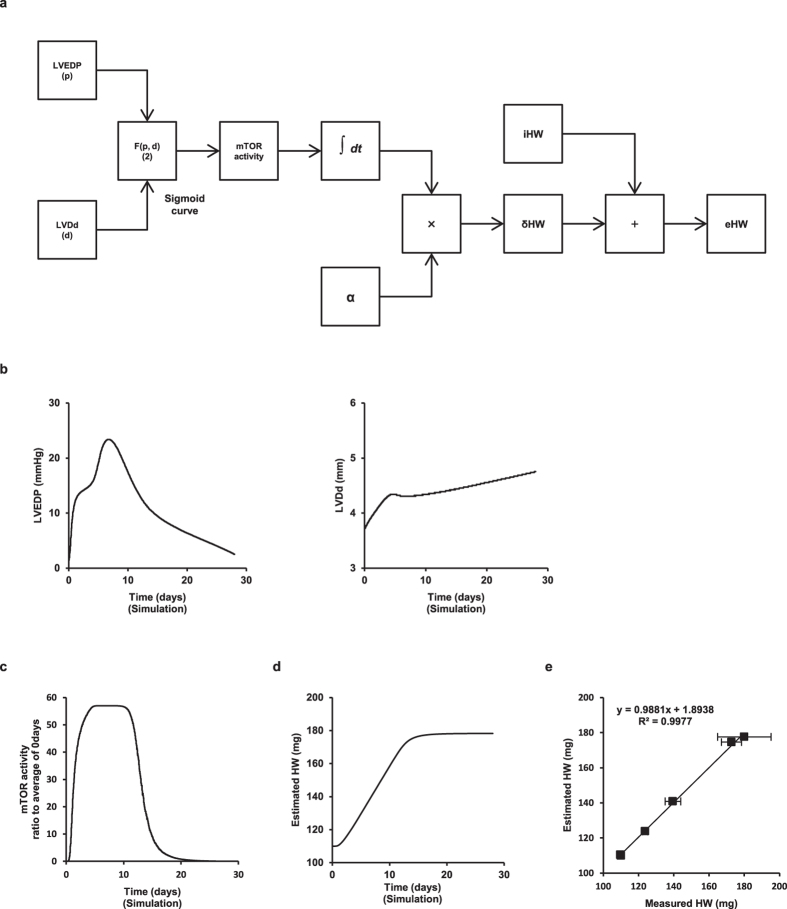
Estimation of eccentric hypertrophy during VO using physiological parameters LVEDP and LVDd. (**a**) Schematic diagram to estimate HW from LVEDP and LVDd. (**b**) LVEDP (left panel) and LVDd (right panel) curves were reproduced with Simulink from resampled data points. (**c**) Estimated mTOR activity during VO. (**d**) Estimated HW during VO when α = 0.001. (**e**) Correlation between measured and estimated HW. Data are shown as the mean ± SEM (n = 4).

Substituting mTOR activity in equation (2) into (1) and σ with d × p/4h yielded eHW as follows:


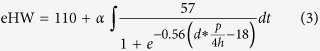


(Figure 8a). Equation 3 indicates that, once the physiological parameters d (LVDd) and p (LVEDP) for the estimation of diastolic wall stress are determined, the progression of eccentric hypertrophy during VO can be accurately estimated. Indeed, data sets based on 100 resampled data points per day of LVEDP and LVDd reproduced the experimentally measured LVEDP and LVDd (Fig. 8b) and allowed accurate estimation of the mTOR activity by equation (2) when the mean wall thickness (h) was approximated by its average value on day 0 (=0.70) (Fig. 8c) as it did not significantly change during VO (Fig. 1e). In addition, we accurately estimated the HW during VO by equation (3) (Fig. 8d), which revealed that eHW was closely correlated with the measured HW when α = 0.001 (Fig. 8e). These results suggested that we could estimate the progression of eccentric hypertrophy in mice from physiological parameters such as LVEDP and LVDd.

## Discussion

Three major findings emerged from the analysis of the VO model at both the physiological and molecular levels: 1) mTOR, whose activity depends on diastolic wall stress, is a pivotal regulator of eccentric hypertrophy during VO; 2) Akt is a major mediator of mTOR activation upon mechanical stretch in diastole; and 3) the rate of eccentric hypertrophy progression during VO is proportional to mTOR activity, allowing accurate estimation of eccentric hypertrophy progression based on LVDd and LVEDP data sets.

To the best of our knowledge, this is the first report on the precise identification of the quantitative relationship between LVEDP as a hemodynamic load and mTOR activity during VO in the heart, and the role of mTOR in eccentric hypertrophy. Some previous studies focused on the role of mTOR in the PO model and revealed that mTOR was activated in the heart in this model^22^. However, currently, there is no evidence that mTOR activity is correlated with systolic pressure or systolic wall stress, although mTOR activity was temporarily increased in the acute phase of a transverse aortic constriction (TAC) model^22^. Aortic constriction *in vivo* increases not only systolic pressure but also LVEDP because an acute rise in afterload in the aortic constriction model inevitably reduces cardiac output and increased LVEDP^31^. Furthermore, there is no doubt that mTOR is activated in the chronic phase of the PO model because LVEDP generally increases in decompensated HF^32^. These interpretations could elucidate why mTOR is also activated in the PO model. We demonstrated that mTOR activity is closely correlated with diastolic wall stress using a VO model, in which no increase in systolic pressure was observed. These results suggested that mTOR activity is directly regulated by LVEDP, and plays a key role in eccentric hypertrophy caused by VO.

We demonstrated that mTOR inhibition induces cardiac atrophy and completely abolishes eccentric hypertrophy in mice treated with temsirolimus during VO when compared to sham-operated mice treated with temsirolimus. Importantly, mTOR inhibition induced cardiac atrophy without upregulation of atrophic genes such as atrogin-1 and muscle RING-finger protein (MuRF)-1, which function as ubiquitin ligases (Supplementary Fig. S6). These results indicated that the mass or size of the heart was determined by the balance between protein synthesis and degradation and that cardiac atrophy occurred when the balance was weighted toward protein degradation under mTOR inhibition even without upregulation of atrophic genes, while eccentric hypertrophy occurred when protein synthesis under VO dominated.

However, the cause of death in mice treated with temsirolimus during VO was not clear because these animals were severely weakened and died shortly after anesthetic administration; therefore, we could not measure the LVEDP for this group. However, we speculated that the low cardiac output from the smaller LV would result in higher LVEDP and be lethal for AVF operated-mice treated with temsirolimus because systolic function, defined by echocardiographically determined LVDs and LVEF, was preserved among all groups.

The role of mTOR in hypertrophy of the skeletal muscle is similar to that in eccentric hypertrophy in the heart^33^,^34^. In the skeletal muscle, Akt is one of the major mediators of mechanical stretch-induced mTOR activation^33^, while other signals including amino acids, Ca^2+^, and phosphatidic acids are also involved^34^. It has been recently reported that the neuronal nitric oxide synthase (nNOS)/Ca^2+^/mTOR pathway is a key pathway in stretch-induced hypertrophy of the skeletal muscle^16^. We found that Akt inhibition by MK-2206 abolished mTOR activation during VO, underscoring its importance as a key mediator of mTOR signaling, although this does not preclude the involvement of other factors.

Meanwhile, the activator for Akt during VO remains to be definitively elucidated, although we identified candidates including IGF, NRG-1, and CTGF. NRG-1 is released from the endothelial cells of the microvessels in the heart^35^, and IGF is released from myocytes in response to mechanical stretch^36^. Because it was technically difficult to investigate the secretion of these growth factors *in vivo* during VO, we measured their mRNA expression in the sub-acute phase (day 1 to 14). Therefore, the contribution of these growth factors to Akt-mTOR activation during VO, especially in the acute phase (1–3 h), is not conclusive. It was previously reported that IGF-1 is rapidly secreted (within 30 min) from myocytes in response to mechanical stretch *in vitro*^36^; we therefore speculated that secretion of these growth factors would precede mRNA upregulation and contribute to the activation of the Akt-mTOR axis in the acute phase (1–3 h). However, further investigation is needed to confirm the role of growth factors in Akt-mTOR activation during VO. Moreover, the K63 ubiquitination of Akt in response to VO may hold a clue for the identification of the activator of the Akt-mTOR axis. Recent reports have indicated that IGF and epidermal growth factors regulate K63 ubiquitination of Akt, leading to its recruitment to the cell membrane and promoting its phosphorylation^26^,^27^. Our results indicating K63 ubiquitination are consistent with the upregulation of IGF-1 or NRG-1 and Akt-mTOR activation. It would be worthwhile to further investigate the role of not only IGF-1 and NRG-1, but also K63 ubiquitination, in Akt-mTOR activation during VO. In addition to IGF and NRG-1, CTGF, which has previously been reported to activate Akt in isolated rat neonatal myocytes, might contribute to Akt-mTOR activation during VO^37^. Besides these growth factors, we investigated the integrin-linked kinase (ILK), focal adhesion kinase (FAK), and G protein-coupled protein receptor (GPCR), but found no clear evidence of their involvement in Akt activation during VO (Supplementary Fig. S7a–c).

As mTOR plays a key role in organ growth and determines the organ size^11^,^19^,^21^, eccentric hypertrophy, which mainly depends on mTOR activity, is analogous to cardiac growth, and mTOR activity physiologically regulates the size of the adult heart in accordance with the individual’s physique and corresponding stressed volume (preload). Apart from its role in cardiac growth, the Akt-mTOR axis plays a key role in exercise-induced hypertrophy^38^,^39^. Exercise induces not only enhanced contractility and increased heart rate, but also reduced arterial resistance and increased stressed volume (preload) with mild increase of LVEDP^40^,^41^. We speculated that the increase in stressed volume and LVEDP during exercise would activate Akt-mTOR, resulting in exercise-induced hypertrophy, even though exercise produces a composite stimulation for the heart as mentioned earlier.

We demonstrated that the HW during VO could be accurately estimated by time integration of mTOR activity based on the evidence that the rate of eccentric hypertrophy progression was proportional to mTOR activity, which underlines the pivotal role of mTOR in eccentric hypertrophy. One reason for which this simple calculation allows the accurate estimation of the HW during VO might be the low proliferative capacity of myocytes. For other organs, a more complex model would be required because the presence of proliferative cells would yield an increase in mass that is independent of the cellular growth resulting from the mTOR activity.

Although we focused on the role of Akt-mTOR in eccentric hypertrophy in this study, the role of Akt-mTOR in cardiac function is also an interesting issue. Akt plays a key role in the pro-survival pathway through various signalings^42^,^43^, and mTORC1 regulates cardiac function and myocyte survival through 4E-BP1 inhibition^44^. Our preliminary data showed that myocyte apoptosis was not evident in eccentric hypertrophy during VO as reported previously^6^ (Supplementary Fig. S8) and therefore we did not further investigate the role of the Akt-mTOR axis in cardiac function in this study; however, it would be of great interest to investigate the role of the Akt-mTOR axis in response to diastolic wall stress in cardiac function in failing myocardium, in which myocyte apoptosis frequently occurs.

In conclusion, the Akt-mTOR axis, which is activated in an LVEDP-dependent manner, plays a pivotal role in eccentric hypertrophy progression. As eccentric hypertrophy is an inherent system of the heart for regulating cardiac output and LVEDP, our findings provide a new mechanistic insight into the adaptive mechanism of the heart.

## Methods

### Mice and arteriovenous fistula (AVF) creation

All procedures involving animals and animal care protocols were approved by the Committee on Ethics of Animal Experiments of the Kyushu University Graduate School of Medical and Pharmaceutical Sciences (A26-053), and were performed in accordance with the Guideline for Animal Experiments of Kyushu University and the Guideline for the Care and Use of Laboratory Animals published by the US National Institutes of Health (revised in 2011). C57BL/6J mice (purchased from Kyudo Co. Ltd., Saga, Japan) were housed in a temperature- and humidity-controlled room and were fed a commercial diet (CRF-1; Oriental Yeast Co. Ltd., Tokyo, Japan) with free access to water. The VO model was generated by creating an AVF as previously described^45^. Briefly, 8- to 10-week-old male mice were anesthetized with a mixture of medetomidine (0.3 mg/kg; Wako Chemicals, Osaka, Japan), midazolam (4 mg/kg; Wako Chemicals), and butorphanol tartrate (5 mg/kg; Wako Chemicals) according to institutional recommendations, or with 1.5–2% isoflurane (Pfizer, New York, NY, USA) plus butorphanol tartrate (5 mg/kg; Wako Chemicals) for experiments within 24 h. The abdominal aorta was clipped and penetrated from the arterial to the venous side with a needle, and the hole on the arterial side was sealed with cyanoacrylate. Before closure of the abdominal incision, the mice were intraperitoneally administered 0.2 mL normal saline to replenish the fluid loss due to hemorrhage. Successful AVF creation was confirmed by observing red oxygenated arterial blood in the vein at the time of surgery and at sacrifice. Control mice underwent sham surgery without AVF creation. Mice were starved for over 12 h before sacrifice.

### Echocardiographic and hemodynamic measurements

Under light anesthesia with 1–2% isoflurane, two-dimensional targeted M-mode images were obtained from the short axis view at the papillary muscle level using a Vevo 2100 ultrasonography system (Visual Sonics, Toronto, Canada). The LVEF was calculated by the following formula that was pre-programmed in the system: LVEF = [(diastolic LV volume—systolic LV volume)/diastolic LV volume) × 100], where LV volume = [(7.0/(2.4 + LV diameter)] × LV diameter^3^. The mean wall thickness was measured as the mean value of the thicknesses of the interventricular septum and the left ventricular posterior wall. Hemodynamics were measured while mice were lightly anesthetized with tribromoethanol-amylene hydrate (2.5% Avertin; 8 μL/g body weight by intraperitoneal injection; Sigma, St. Louis, MO, USA). A 1.4-Fr micro-manometer-tipped catheter (Millar Instruments Inc., Houston, TX, USA) was inserted into the right carotid artery and advanced into the left ventricle for pressure measurement. Diastolic wall stress (σ) was calculated from the LV diameter in diastole (LVDd) (d), mean wall thickness (h) as measured by echocardiography, and LVEDP (p) according to the formula σ = d × p/4 h, assuming a spherical geometry. After the measurements, the mice were euthanized with an overdose of sodium phenobarbital.

### Western blotting

Frozen myocardium tissue samples were homogenized in radioimmunoprecipitation assay (RIPA) buffer (Thermo Fisher Scientific, Waltham, MA, USA) containing a protease inhibitor cocktail (Roche Diagnostics, Indianapolis, IN, USA), 1 mM NaF, and 0.1 mM Na_3_VO_4_. Equal amounts of protein (20 μg per lane) were separated by sodium dodecyl sulfate-polyacrylamide gel electrophoresis (SDS-PAGE) and then electrophoretically transferred to a nitrocellulose membrane using a Trans-Blot apparatus (Bio-Rad, Hercules, CA, USA). After blocking for 1 h with skim milk in Tris-buffered saline with 0.1% Tween 20, the membrane was incubated with primary antibody at 4 °C overnight, followed by incubation with the appropriate secondary antibody at room temperature for 1 h. Primary antibodies from Cell Signaling Technology (Danvers, MA, USA) against the following proteins were used: ribosomal protein S6 (#2217), phospho-S6^Ser235/236^ (#4858), phospho-S6^240/244^ (#5364), eukaryotic translation initiation factor 4E binding protein (4E-BP; #9644), phospho-4E-BP^Thr37/46^ (#2855), Akt (#9272), phospho-Akt^Ser473^ (#4060), proline-rich Akt substrate 40 kDa (PRAS40; #2691), phospho-PRAS^Thr246^ (#13175), K63-linkage specific ubiquitin (#5621), cleaved caspase-3 (#9661), and poly ADP ribose polymerase (PARP; #9542). An antibody against glyceraldehyde-3-phosphate dehydrogenase (GADPH; sc-32233; Santa Cruz Biotechnology, Santa Cruz, CA, USA) was used as a control.

### Immunoprecipitation

Myocardium tissue homogenates were immunoprecipitated with Akt antibody (#9272) in RIPA buffer containing a protease inhibitor cocktail, 1 mM NaF, 0.1 mM Na_3_VO_4_, and 20 μM PR-619 at 4 °C for 1 h. Protein G-sepharose beads (GE Healthcare, Pittsburgh, PA, USA) were added to the mixture and incubated at 4 °C for 1 h. The mixture was centrifuged and washed three times. An equivalent volume of sample buffer was added to each sample. After vortexing and boiling, equal volumes of the samples were separated by SDS-PAGE, followed by immunoblotting as described above.

### Administration of temsirolimus and Akt inhibitor to mice

Osmotic pumps (ALZET, Cupertino, CA, USA) filled with temsirolimus solution (Pfizer, New York, NY, USA) were subcutaneously implanted into the mice. The Akt inhibitor MK-2206 (Active Biochemicals Co., Hong Kong) was dissolved in 30% captisol (ChemScene, Monmouth Junction, NJ, USA) and orally administered. Control mice were administered the vehicle.

### Quantitative reverse transcription PCR (RT-qPCR)

Total RNA was extracted with the RNeasy Mini kit (Qiagen, Valencia, CA, USA) and converted to cDNA using a ReverTra Ace kit (Toyobo, Tokyo, Japan). The relative expression of each target gene was measured by RT-qPCR from cDNA equivalent to 10 ng RNA using 12 pmol of each primer in a 30-μL reaction volume. The reactions were run in an AB7500 Fast Real-Time PCR system (Life Technologies, Rockville, MD, USA). The *18S* ribosomal RNA gene was used as an internal control. The forward and reverse primer sequences, respectively, were as follows: IGF-1, CTGGACCAGAGACCCTTTGC and GGACGGGGACTTCTGAGTCTT; IGF-2, GTG CTGCATCGCTGCTTAC and ACGTCCCTCTCGGACTTGG; NRG-1, GGCATCTGTATCGCCCTGTTG and TGAGGGCCATTCGCTATGTTC; CTGF, TGCAGACTGGAGAAGCAGAG and CGATTTTAGGTGTCCGGATG; PDGF-A, GCGACTCTTGGAGATAGACTCCGTA and CGTAAATGACCGTCCTGGTCTTG; PDGF-B, TGCTGAGCGACCACTCCATC and CTCGGGTCATGTTCAAGTCCA; PDGF-C, ACCCGGATTCTGCATCCACTAC and GTCCACCTGCCATCGATCTG; PDGF-D, GCGGAACTGTCAACTGGAAGTC and CTCTTGAAATGTCCAGGCTCAAAC; atrogin-1, AACATGTGGGTGTATCGGATGG and TGATGTTCAGTTGTAAGCACACAGG; MuRF-1, TGTCTCACGTGTGAGGTGCCTA and CACCAGCATGGAGATGCAGTTAC; and 18S, TTCTGGCCAACGGTCTAGACAAC and CCAGTGGTCTTGGTGTGCTGA.

### Computational simulation

The Simulink software (MathWorks, Natick, MA, USA) was used to perform a simulation with the time series of mTOR activity, LVEDP, and LVDd. We linear- or curve-fitted the measured mTOR activity, LVDd and LVEDP data using the Excel software (Microsoft, Redmond, WA, USA), and the fitted curves were resampled at 100 points/day using the online software Graphcel v1.11 to acquire a data set for each parameter that was then used as input data for the simulation model.

### Statistical analysis

Data are shown as the mean ± SEM. To compare the treatment groups, we used one-way analysis of variance (one-way ANOVA) followed by Tukey’s or Dunnett’s post-hoc test. Survival analysis was performed using the Kaplan-Meier method and between-group differences in survival were evaluated with the log-rank test. *P* < 0.05 was considered significant. The JMP9 software (SAS, Cary, NC, USA) was used for the statistical analysis.

## Additional Information

**How to cite this article**: Ikeda, M. *et al.* The Akt-mTOR axis is a pivotal regulator of eccentric hypertrophy during volume overload. *Sci. Rep.* **5**, 15881; doi: 10.1038/srep15881 (2015).

## Supplementary Material

Supplementary Information
