# Supplementary material for: The Akt-mTOR axis is a pivotal regulator of eccentric hypertrophy during volume overload
Source: Sci Rep. 2015 Oct 30;5:15881. doi: 10.1038/srep15881 (PMC4626834; doi:10.1038/srep15881)
Supplement: Supplementary Information [file srep15881-s1.pdf]

Supplementary Information for:

The Akt-mTOR axis is a pivotal regulator of eccentric hypertrophy during volume overload

Masataka Ikeda*, Tomomi Ide*, Takeo Fujino, Yuka Matsuo, Shinobu Arai, Keita Saku, Takamori Kakino, Yasuhiro Oga, Akiko Nishizaki, and Kenji Sunagawa

Department of Cardiovascular Medicine, Graduate School of Medical Sciences, Kyushu University, Fukuoka, Japan

*To whom correspondence should be addressed:
E-mail: ikeda-m@cardiol.med.kyushu-u.ac.jp (M.I.),
tomomi_i@cardiol.med.kyushu-u.ac.jp (T.I.)

Supplementary Figure S1

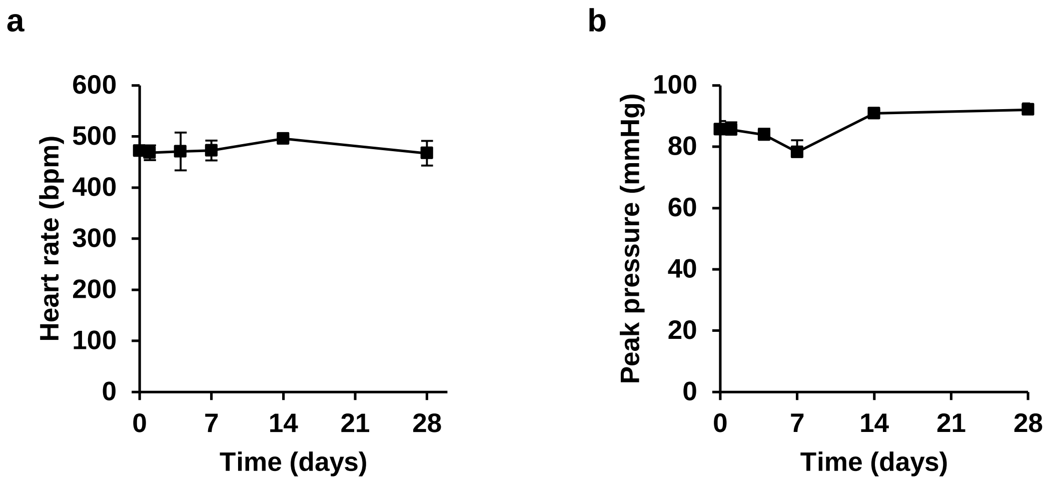

Supplementary Figure S1: Physiological parameters during the 4-week time course of VO. (**a**) Heart rate. (**b**) Peak pressure. Data are shown as the mean±SEM (n=4).

Supplementary Figure S2

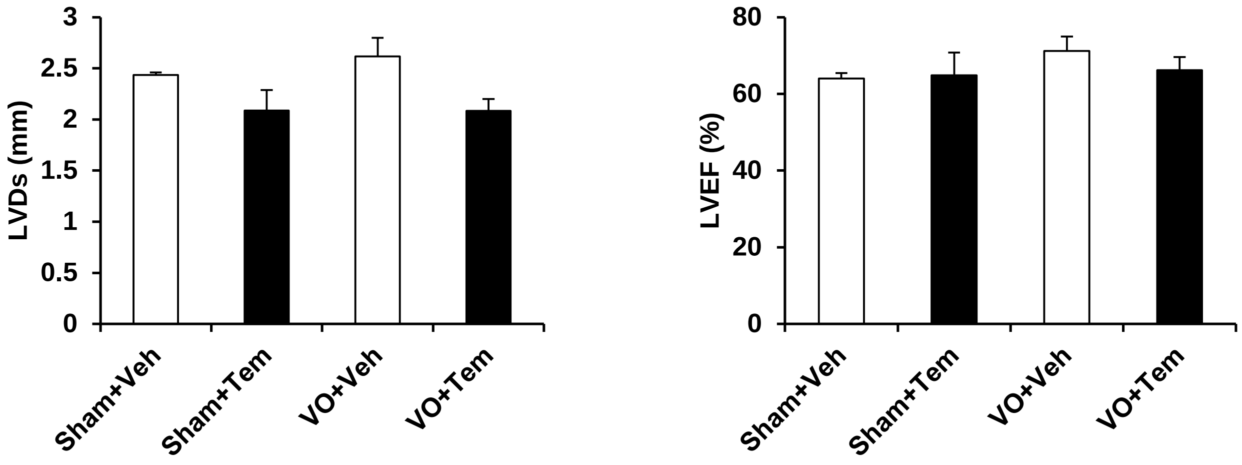

Supplementary Figure S2: Effect of mTOR inhibition on cardiac function on day 3 after VO creation. LVDs (left panel) and LVEF (right panel) on day 3 of VO (n=3–4).

Supplementary Figure S3

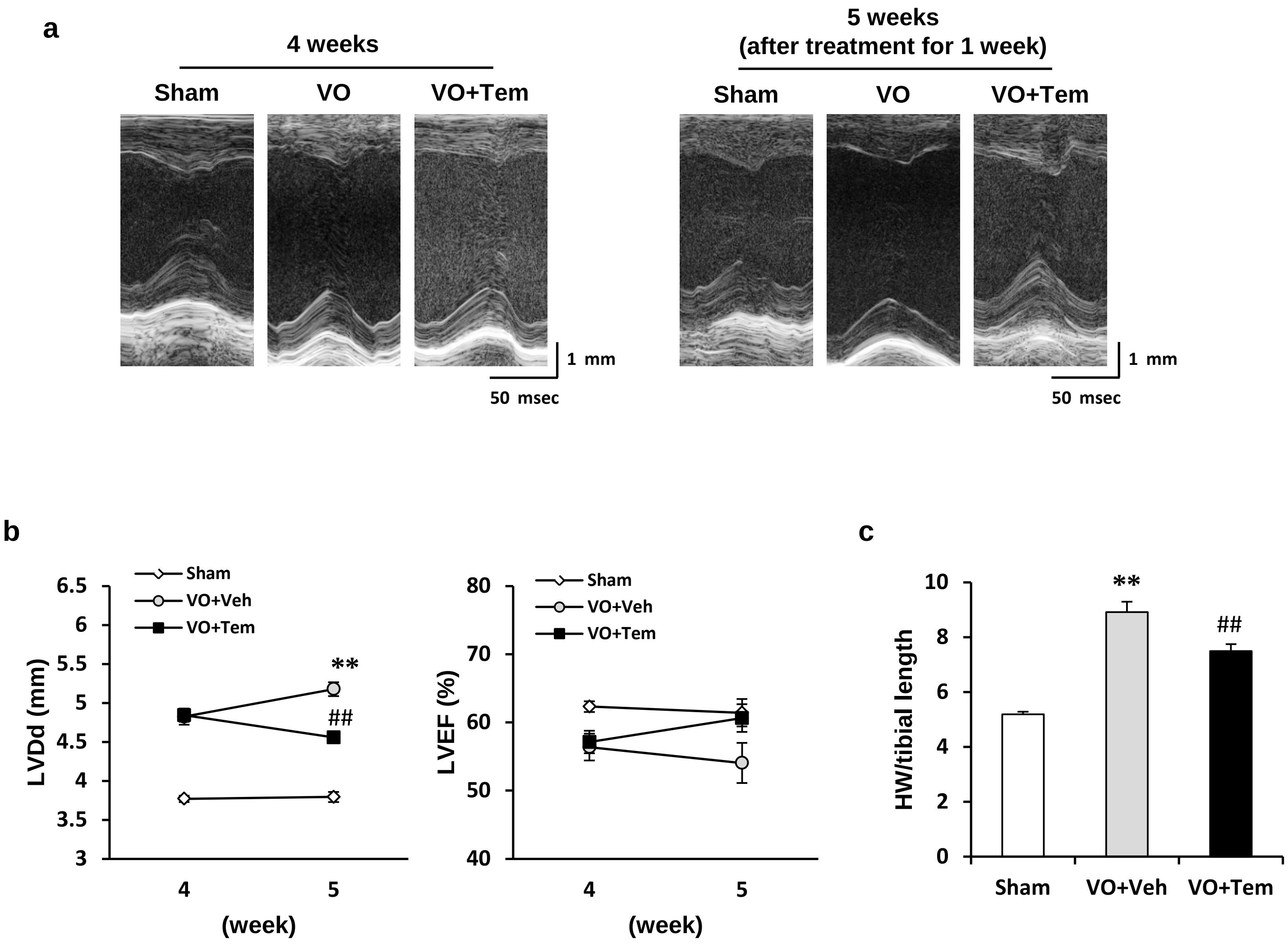

Supplementary Figure S3: Effect of mTOR inhibition on established eccentric hypertrophy at 4 weeks after VO creation. (**a**) Echocardiogram at 4 weeks after AVF creation or sham operation (left panel), VO mice were randomized into two groups and treated with vehicle (Veh) or temsirolimus (Tem) for 1 week (right panel). (**b**) LVDd (left panel) and LVEF (right panel) before and after Tem treatment for 1 week, Sham, n=6; VO + Veh, n=13; VO + Tem, n=13. (**c**) HW per tibial length after 1 week of Tem treatment, Sham, n=6; VO + Veh, n=13; VO + Tem, n=13. Data are shown as the mean±SEM. $^{**}P$ < 0.01 vs. sham, $^{\#}P$ < 0.05, $^{\#\#}P$ < 0.01 vs. VO + Veh (one-way ANOVA).

Supplementary Figure S4

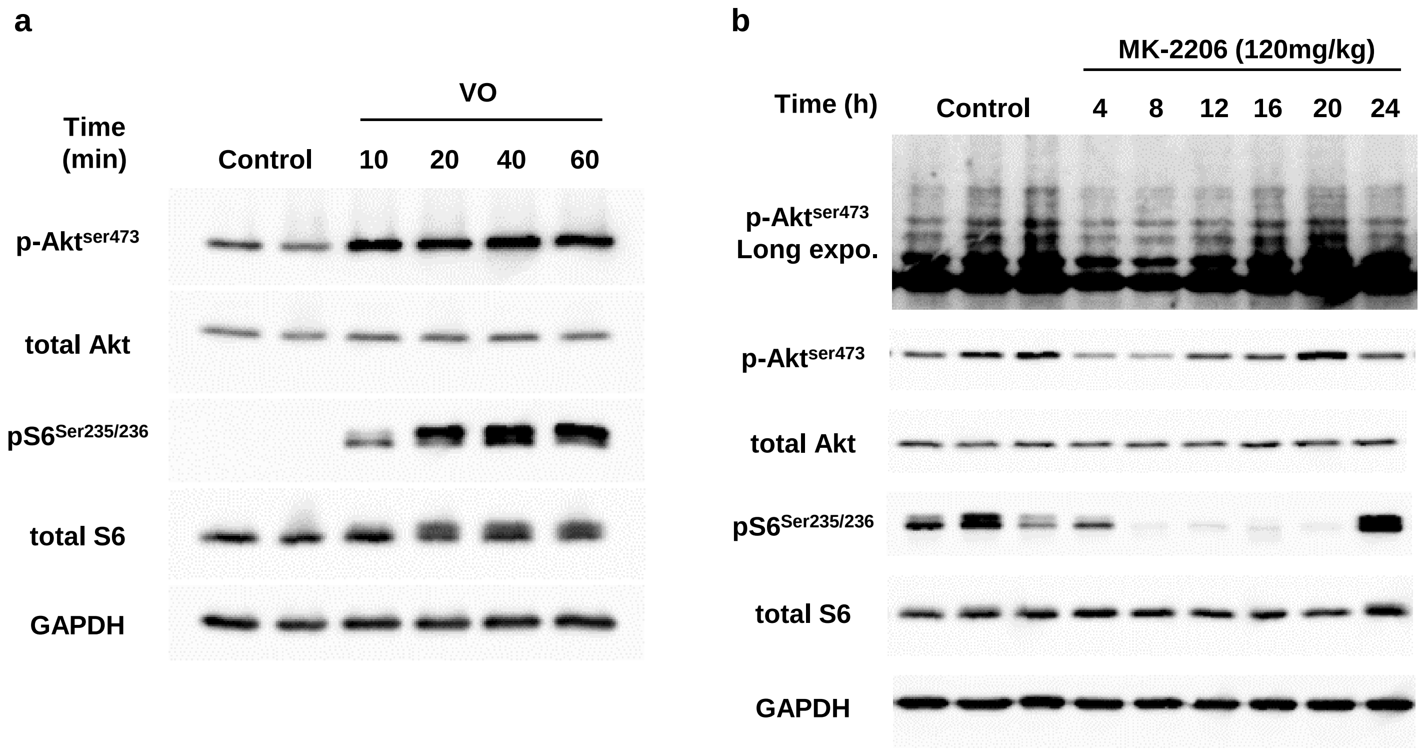

Supplementary Figure S4: Western blot probed for total and phosphorylated Akt and S6. (**a**) Sixty-minute time course after arteriovenous fistula creation (AVF). (**b**) Twenty-four-hour time course after administration of the allosteric Akt inhibitor MK-2206 (with long exposure time in the upper panel). GAPDH was used as a loading control.

Supplementary Figure S5

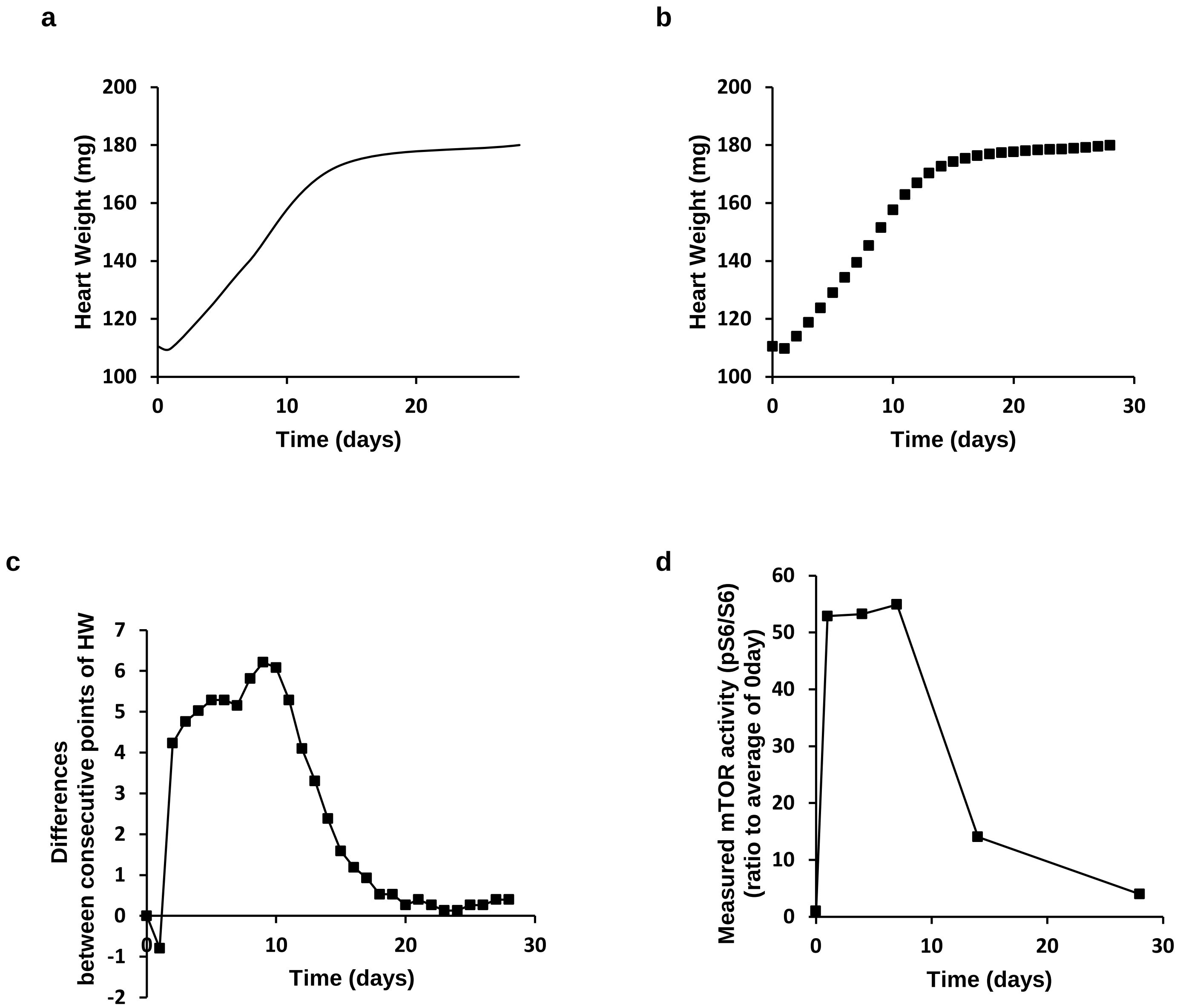

Supplementary Figure S5: Mathematical analysis of HW trends and mTOR activity during VO. (**a**) Fitted curve of HW during VO. (**b**) Twenty-eight resampled points (equivalent to each day) from the fitted curve. (**c**) Plots of the differences between consecutive resampled points corresponding to the rate of eccentric hypertrophy progression. (**d**) Plots of the average mTOR activity at day 0, 1, 4, 7, 14, and 28 (n=2).

Supplementary Figure S6

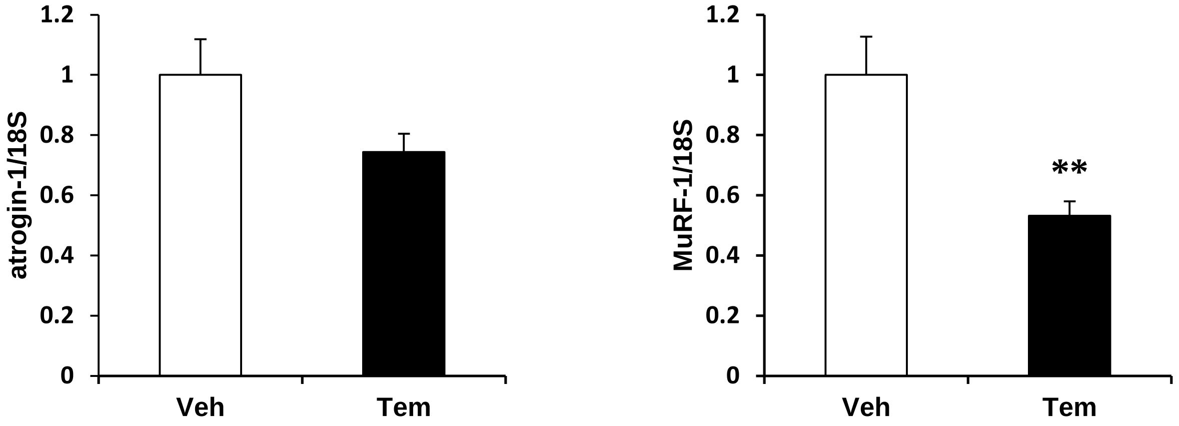

Supplementary Figure S6: mRNA expression of atrophic genes (atrogin-1 and MuRF-1) in the heart at 3 days after treatment with temsirolimus (5 mg/kg/day). Data are shown as the mean±SEM (n=4–6). ***P* < 0.01 vs. Veh.

Supplementary Figure S7

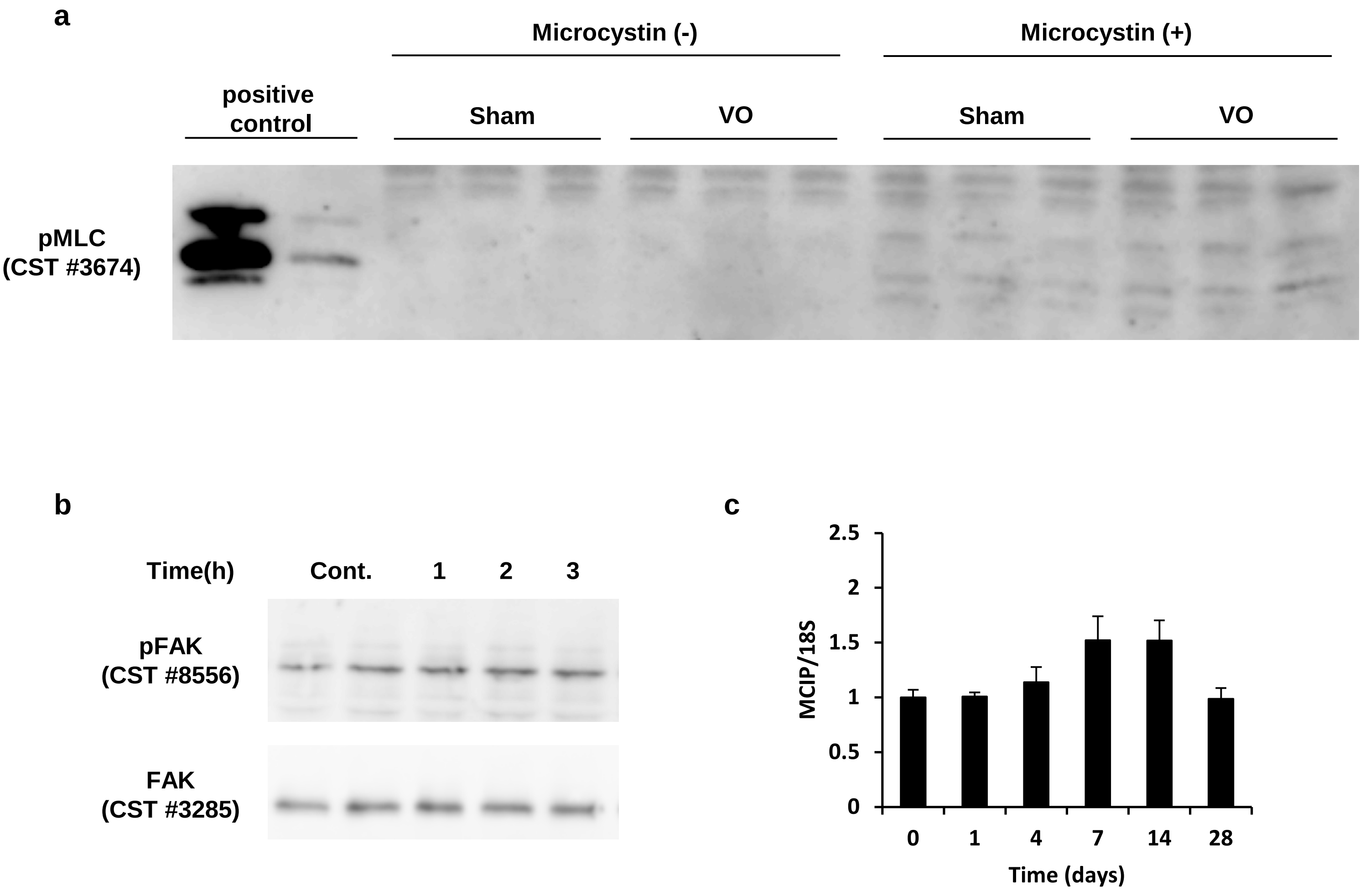

Supplementary Figure S7: Involvement of integrin-linked kinase (ILK), focal adhesion kinase (FAK), and G protein-coupled receptors (GPCR) during volume overload (VO). (**a**) Western blot of cell lysates obtained from heart at day 3 after AVF creation, probed for phosphorylated myosin light chain (MLC) using protein extraction buffer with or without microcystin (5 μM). The positive control consisted of total protein derived from rat vascular smooth muscle cell (VSMC). (**b**) Western blot of cell lysates obtained from heart at 0–3 h after AVF creation probed for total and phosphorylated FAK. (**c**) mRNA expression of MCIP (MCIP; modulatory calcineurin-interacting protein) during VO, which is regulated by GPCR-NFAT (NFAT; nuclear factor of activated T-cells).

Supplementary Figure S8

a

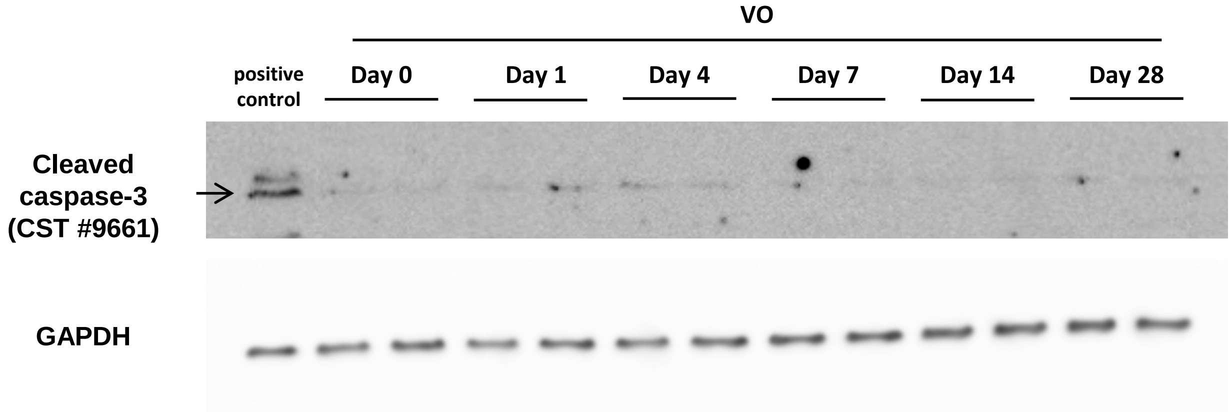

b

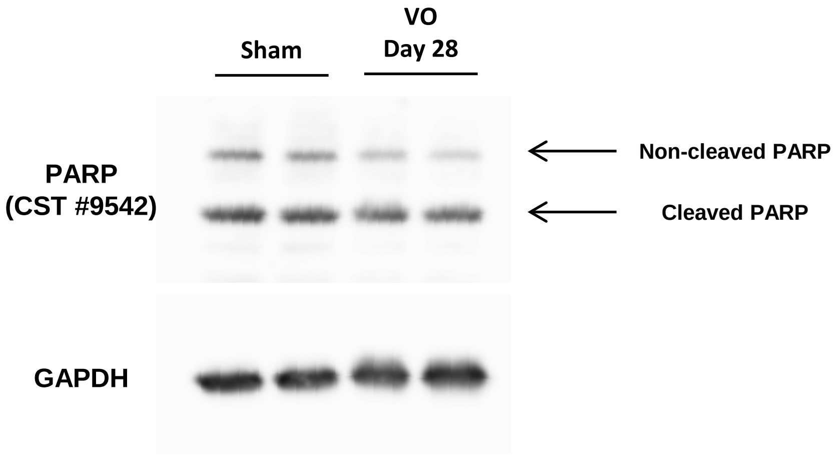

Supplementary Figure S8: Western blot analysis for apoptosis-related signals. (**a**) Cleaved caspase-3 during VO. The positive control consisted of total protein derived from RAW264.7, stimulated by palmitate. (**b**) PARP (PARP; poly ADP ribose polymerase) and cleaved PARP at day 28.
